# Clinical outcomes of carbon ion radiotherapy with concurrent chemotherapy for locally advanced uterine cervical adenocarcinoma in a phase 1/2 clinical trial (Protocol 1001)

**DOI:** 10.1002/cam4.1305

**Published:** 2018-01-17

**Authors:** Noriyuki Okonogi, Masaru Wakatsuki, Shingo Kato, Kumiko Karasawa, Hiroki Kiyohara, Shintaro Shiba, Daijiro Kobayashi, Takashi Nakano, Tadashi Kamada, Makio Shozu, the Working Group of Gynecological Tumors

**Affiliations:** ^1^ National Institute of Radiological Sciences Hospital National Institutes for Quantum and Radiological Science and Technology Chiba Japan; ^2^ Department of Radiology Jichi Medical University Tochigi Japan; ^3^ Department of Radiation Oncology Saitama Medical University International Medical Center Saitama Japan; ^4^ Department of Radiation Oncology Tokyo Women's Medical University School of Medicine Tokyo Japan; ^5^ Department of Radiation Oncology Maebashi Red Cross Hospital Gunma Japan; ^6^ Department of Radiation Oncology Gunma University Graduate School of Medicine Gunma Japan; ^7^ Department of Reproductive Medicine Chiba University Graduate School of Medicine Chiba Japan

**Keywords:** Adenocarcinoma, carbon ion radiotherapy, cisplatin, concurrent chemoradiotherapy, uterine cervical cancer

## Abstract

We conducted a phase 1/2 study to evaluate the efficacy and safety of carbon ion radiotherapy (C‐ion RT) with concurrent chemotherapy for locally advanced uterine cervical adenocarcinoma. Thirty‐three patients were enrolled between April 2010 and March 2014. Treatment consisted of C‐ion RT with concurrent weekly cisplatin at a dose of 40 mg/m^2^. In the phase 1 component, the total dose was escalated from 68.0 Gy (relative biological effectiveness [RBE]) to 74.4 Gy (RBE) to determine the maximum tolerated dose of C‐ion RT. In the phase 2 component, the efficacy and safety of C‐ion RT with concurrent chemotherapy were evaluated using the dose determined in the phase 1 component. The median follow‐up duration was 30 months. Two patients did not receive chemotherapy because of anemia or leukocytopenia immediately prior to commencing treatment; 31 patients were analyzed. None of the patients developed dose‐limiting toxicities. The recommended dose (RD) was determined to be 74.4 Gy (RBE). In the phase 2 component, two patients developed Grade 3–4 toxicities in the gastrointestinal tract, due to repeated laser coagulation or peritonitis caused by appendicitis. In the patients treated with the RD, the 2‐year local control, progression‐free survival, and overall survival rates were 71%, 56%, and 88%, respectively. C‐ion RT with concurrent weekly cisplatin was well tolerated in patients with locally advanced uterine cervical adenocarcinoma. Our findings support further investigations into the efficacy of this strategy.

## Introduction

Uterine cervical cancer remains one of the most common cancers in women worldwide 1. The global incidence of cervical cancer in 2012 was 528,000, with 266,000 deaths reported per annum and approximately 85% of cases occurring in developing countries 2. The incidence and mortality rates of squamous cell carcinoma are declining. However, the incidence of adeno/adenosquamous carcinoma is rising in many countries, despite being relatively uncommon histological subtypes of cervical cancer 1, 3.

The primary treatment for early‐stage cervical cancer is surgery or radiotherapy (RT). Concurrent chemoradiotherapy (CCRT) is considered the primary treatment of choice for Stage IB2‐IVA uterine cervical squamous cell carcinoma based on randomized clinical trials and meta‐analyses 4, 5, 6, 7, 8. Recent studies have shown that CCRT and image‐guided brachytherapy improve the clinical outcome of patients with cervical carcinoma, especially squamous cell carcinoma 9, 10, 11. Conversely, few studies have investigated treatment options for adenocarcinomas; these are typically treated in a similar manner to squamous cell carcinomas 12, 13. However, uterine cervical adenocarcinomas are more radioresistant than squamous cell carcinomas 12, 14, with poorer local control (LC) and overall survival (OS). Therefore, a more aggressive approach is needed for locally advanced uterine cervical adenocarcinomas.

Carbon‐ion (C‐ion) RT was adopted at the National Institute of Radiological Sciences in 1994. C‐ion beams have improved dose localization properties, which can potentially produce great effects on tumors while minimizing normal tissue damage. C‐ion beams also possess a biological advantage due to their high‐linear energy transfer in the Bragg peak 15, 16. Several phase 1/2 and phase 2 studies^17^, ^18^, ^19^ of C‐ion RT have been conducted in patients with several types of malignancies, including strategies that comprise shorter treatment fractions, in shorter overall treatment time, compared to standard therapy. In a previous clinical trial 18, we investigated the treatment outcomes of C‐ion RT for locally advanced uterine cervical adenocarcinomas. Dose escalation of C‐ion RT was achieved without severe toxicity in all but one case. The 5‐year LC and OS rates were 55% and 38%, respectively 15. The LC rate was higher than those of conventional studies 14, 20, 21, 22, but it was still less than satisfactory. The 2‐ and 5‐year distant metastasis (DM) rates were 49% and 65%, respectively. Thus, to improve LC and reduce the rate of DM, concurrent chemotherapy with C‐ion RT (chemo‐C‐ion RT) was considered for locally advanced uterine cervical adenocarcinoma.

The feasibility of chemo‐C‐ion RT for the pelvic region has yet to be determined. Therefore, we conducted a phase 1/2 clinical trial (protocol 1001) between April 2010 and March 2014 to evaluate the efficacy and safety of chemo‐C‐ion RT for locally advanced uterine cervical adenocarcinoma.

## Materials and Methods

### Patients

Patients were enrolled provided they had previously untreated Stage IIB, III, or IVA uterine cervical adeno/adenosquamous carcinoma without rectal invasion according to *Union for International Cancer Control TNM Classification of Malignant Tumors*, 7th edition. The primary tumor had to be grossly measurable. Patients with enlarged pelvic lymph nodes were included. However, patients with para‐aortic lymph nodes of ≥1.0 cm in minimum diameter on computed tomography (CT) images were excluded. All patients (1) were between 20 and 70 years of age, (2) had an Eastern Cooperative Oncology Group performance status of 0–2, and (3) had no prior treatment with pelvic RT or systemic therapy. Other eligibility criteria included adequate bone marrow (hemoglobin level, ≥10.0 g/dL; leukocyte count, ≥3000/mL; and platelet count, ≥100,000/mL), and renal and hepatic (serum creatinine level, <1.5 mg/dL; bilirubin level, <1.5 mg/mL; and aspartate/alanine aminotransferase level, <100 IU/dL) function. Patients were excluded if they had a severe pelvic infection or psychological illness, severe diabetes mellitus, or active double cancer.

CT scans of the abdomen and pelvis, magnetic resonance imaging (MRI) of the pelvis, and fludeoxyglucose (^18^F) positron emission tomography‐CT scans for accurate staging were performed for all the patients. Tumor size was assessed by pelvic examination and MRI. The dimensions of the cervical tumors were measured using T2‐weighted MRI images 23. Working group pathologists reviewed the tumor specimens that were biopsied from local tumors prior to treatment. All participants were explained standard treatment including brachytherapy, and then provided informed written consent of this study. Approval was obtained from our Institutional Review Board.

### Carbon‐ion radiotherapy

Patients were positioned in customized cradles and immobilized with a low‐temperature thermoplastic sheet. A set of 5‐mm‐thick CT images was taken for three‐dimensional treatment planning using HIPLAN software or Xio‐N2 (National Institute of Radiological Sciences, Chiba, Japan). Patients were administered C‐ion RT daily for 4 days/week (Tuesday through to Friday). The radiation dose was calculated for the target volume and surrounding normal structures and was expressed in Gy (relative biological effectiveness [RBE]), which was defined as the physical doses multiplied by the RBE of the C‐ions 16, 24.

### Patient preparation

At every treatment session, the patient was positioned on the treatment couch with the immobilization devices, and the patient's position was verified using a computer‐aided online positioning system. Digital orthogonal X‐ray images were taken and transferred to the positioning computer. The positioning images were compared with reference images that were digitally reconstructed from CT scans. If the difference in positioning was >2 mm, the treatment couch was adjusted until an acceptable position was attained. In addition, if gas was detected in the rectum in digital orthogonal X‐ray images, patients were given enemas to clear out any stool. To minimize internal motion of the uterine cervix, 100–150 mL of normal saline was infused into the bladder and vaginal packing was done tightly at each treatment session. In addition, the cotton for vaginal packing was soaked in a contrast medium so that the surface of the uterine cervix could be visualized by X‐ray images at the treatment sessions for the last eight fractions. Patients were encouraged to take laxatives to prevent constipation during the treatment period.

### Target delineation and margin definition

**Figure 1:**
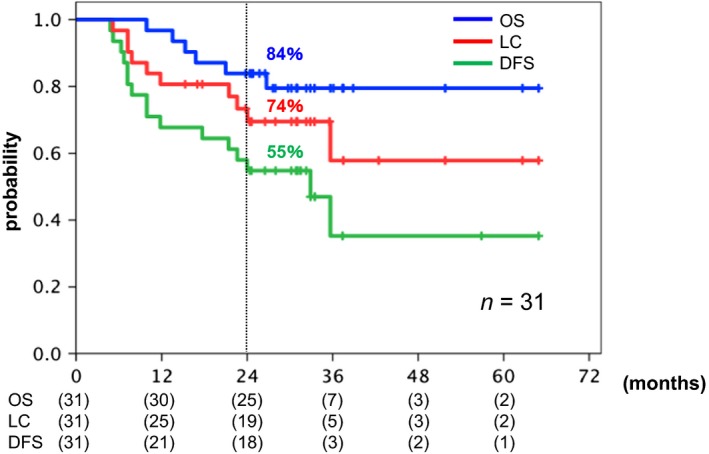
Kaplan–Meier curves of overall survival (OS; blue), local control (LC; red), and disease‐free survival (DFS; green) for all 31 patients combined. Number at risk is shown below the figure.

Treatment consisted of whole pelvic irradiation and local boost. The gross tumor volume was defined by MRI findings and clinical examination immediately prior to each planning session. The clinical target volume (CTV) of whole pelvic irradiation included all areas of gross and potentially microscopic disease, which consisted of the primary site (gross tumor volume, whole uterus, parametrium, and at least the upper half of the vagina and ovaries) and whole pelvic node region (common, internal, and external iliac, obturator, and presacral node regions) (CTV‐1). The first planning target volume (PTV‐1) included CTV‐1 plus a 5‐mm safety margin for positioning uncertainty and the uterus plus a 10‐mm safety margin for intra‐ and inter‐movement. PTV‐1 was covered by ≥90% of the prescribed dose. After completion of whole pelvic irradiation, the CTV included the primary site and swollen lymph nodes (CTV‐2). A 5–10 mm margin was added to CTV‐2 to produce PTV‐2. Finally, the CTV was reduced to include only the gross tumor volume (CTV‐3). A 3‐mm margin was added to CTV‐3 to produce PTV‐3. If PTV‐1 and PTV‐2 overlapped normal tissue structures (e.g., the rectum, sigmoid colon, bladder, and small bowel in the pelvis), priority was given to the PTV coverage. However, the gastrointestinal (GI) tract was excluded from PTV‐3 to limit the dose to the GI tract to a maximum of 60.0 Gy (RBE). The dose to PTV‐1 was fixed at 36.0 Gy (RBE) (3.0 Gy [RBE] per fraction for 12 fractions) in this protocol. With respect to PTV‐2 and PTV‐3, 4 fractions were for PTV‐2 and an additional 4 fractions were for PTV‐3. The dose for PTV‐2 and PTV‐3 was initiated with a fraction dose of 4.0 Gy (RBE) that was escalated in increments to 4.4 or 4.8 Gy (RBE). Thus, the total dose to the cervical tumor was 68.0, 71.2, or 74.4 Gy (RBE) in 20 fractions. Isodose curves of C‐ion RT are shown in FigureS1. Considering the findings of our previous study 18, no further dose increases were planned because they would exceed the dose constraint of the surrounding normal tissue.

### Chemotherapy

Five courses of weekly cisplatin (40 mg/m^2^) were administered during the C‐ion RT treatment period, with the first course administered on Day 1. Chemotherapy was discontinued if (1) the patient developed a Grade ≥3 hematological toxicity, (2) serum creatinine levels were ≥1.5 mg/dL, or (3) aspartate/alanine aminotransferase levels were ≥100 IU/dL. Chemotherapy was also discontinued if the patient developed a Grade ≥3 complication in the GI tract or urinary system.

### Study design

The purpose of the phase 1 component was to determine the maximum tolerated dose of C‐ion RT within the dose range of 68.0–74.4 Gy (RBE) with concurrent chemotherapy at a dose of 40 mg/m^2^. Dose escalation of C‐ion RT was scheduled according to the frequency of dose‐limiting toxicity (DLT), evaluated for 6 months after commencing treatment. DLTs were defined as Grade ≥3 non‐hematological toxicities or Grade 4 hematological toxicities. Three patients were enrolled at each dose level. The dose of C‐ion RT was considered safe if DLTs did not occur in any of the three patients enrolled at each dose level. If one of the three patients enrolled at each dose level developed DLT, then an additional three patients were enrolled. The purpose of the phase 2 component was to evaluate the late toxicities and efficacy using the dose determined in the phase 1 component. Treatment outcomes were measured in terms of response rates 6 months after commencing treatment, LC, disease‐free survival (DFS), and OS 2 years after commencing treatment.

### Efficacy and safety evaluation

Following treatment, patients were followed up every 1–3 months for the first 2 years and every 3–6 months thereafter. Acute toxicity was graded according to the Common Terminology Criteria for Adverse Events (version 4.0) 25, with the highest toxicities occurring within 6 months of commencing treatment. Late toxicity was graded according to the Radiation Therapy Oncology Group/European Organization for Research and Treatment of Cancer Late Radiation Morbidity Scoring Scheme 26. Tumor response was defined using MRI according to the Response Evaluation Criteria in Solid Tumors (version 1.1) 27. Recurrences were detected by physical examination, CT, MRI, positron emission tomography, and/or biopsy.

### Statistical analyses

LC, DFS, and OS curves were plotted using the Kaplan–Meier method. Log‐rank, Mann–Whitney U, and Chi‐square tests were performed using Statistical Package for the Social Sciences for Macintosh, software version 23.0 (IBM Inc., Armonk, NY, USA). A *P *<* *0.05 was considered statistically significant.

## Results

Of the 33 patients enrolled in this study, 27 had cervical adenocarcinoma and 6 had cervical adenosquamous carcinoma. One patient had severe anemia caused by genital bleeding and a second had a leukocyte count of <3000/mL immediately prior to treatment. These two patients were not administrated chemotherapy, and from discussions of the Working Group of Gynecological Tumors, were excluded from further analysis. Thus, a total of 31 patients with cervical adenocarcinoma were analyzed in this study. All 31 patients completed the treatment schedule. The median number of cisplatin administrations was 5 (range, 3–5). The median overall treatment and follow‐up durations were 34 (range, 30–36) days and 30 (range, 10–65) months, respectively. The patient characteristics are summarized in Table 1.

**Table 1:** Patient characteristics

Characteristics	No. of patients enrolled (No. of patients analyzed)
Cases of uterine cervix	33 (31)
Age [median], years	26–70 [47] (26–70 [47])
Histology	
Mucinous adenocarcinoma	17 (17)
Endometrioid adenocarcinoma	7 (6)
Clear cell carcinoma	3 (3)
Adenosquamous carcinoma	6 (5)
UICC TNM stage	
II B	20 (19)
III B	10 (9)
IVA	3 (3)
Tumor size [median], cm	3.0–9.7 [5.2] (3.0–9.7 [5.4])
<5 cm	13 (12)
≤5 cm to >7 cm	12 (11)
≤7 cm	8 (8)
Pelvic LN metastasis	
Yes	14 (12)
No	19 (19)
Dose of C‐ion RT	
68.0 Gy (RBE) in 20 fractions	3 (3)
71.2 Gy (RBE) in 20 fractions	3 (3)
74.4 Gy (RBE) in 20 fractions	27 (25)
No. of weekly CDDP administration	
0 time	2 (0)
3 times	1 (1)
4 times	6 (6)
5 times	24 (24)

UICC, Union for International Cancer Control; LN, Lymph node; C‐ion RT, carbon‐ion radiotherapy; RBE, elative biological effectiveness; CDDP, cisplatin.

### Toxicities

The observed acute toxicities are listed in Table 2A and Table**** 2B. None of the patients developed DLTs in the phase 1 component. Although the maximum tolerated dose was not determined within this dose range, the decision was taken to stop further dose escalation based on the findings of our previous study 18. Finally, the recommended dose (RD) for the phase 2 component was determined to be 74.4 Gy (RBE) in 20 fractions with concurrent weekly cisplatin at a dose of 40 mg/m^2^. With the exception of one patient who developed Grade 3 nausea, none developed severe acute toxicities in the phase 2 component.

**Table 2:** (A) Acute non‐hematological, (B) acute hematological, and (C) late non‐hematological toxicities

		LGIGrade					GUGrade					Nausea/VomitingGrade				
Dose (Phase)	No.	0	1	2	3	4	0	1	2	3	4	0	1	2	3	4
(A)																
68.0 Gy (Phase 1)	3	2	1	0	0	0	3	0	0	0	0	0	3	0	0	0
71.2 Gy (Phase 1)	3	2	1	0	0	0	2	1	0	0	0	0	3	0	0	0
74.4 Gy (Phase 1)	3	3	0	0	0	0	3	0	0	0	0	2	1	0	0	0
74.4 Gy (Phase 2)	22	3	16	3	0	0	19	3	0	0	0	7	9	5	1	0

		Neutrophil decreased Grade					Hemoglobin decreased Grade					Platelet decreased Grade				
Dose (Phase)	No.	0	1	2	3	4	0	1	2	3	4	0	1	2	3	4
(B)																
68.0 Gy (Phase 1)	3	1	0	1	1	0	1	1	1	0	0	2	1	0	0	0
71.2 Gy (Phase 1)	3	1	0	2	0	0	0	2	1	0	0	3	0	0	0	0
74.4 Gy (Phase 1)	3	1	0	2	0	0	2	0	1	0	0	3	0	0	0	0
74.4 Gy (Phase 2)	22	18	1	2	1	0	2	16	4	0	0	18	2	2	0	0

		LGI Grade					GU Grade				
Dose (Phase)	No.	0	1	2	3	4	0	1	2	3	4
(C)											
68.0 Gy (Phase 1)	3	1	2	0	0	0	2	1	0	0	0
71.2 Gy (Phase 1)	3	2	1	0	0	0	2	1	0	0	0
74.4 Gy (Phase 1)	3	2	1	0	0	0	2	0	1	0	0
74.4 Gy (Phase 2)	22	5	11	4	1	1	15	4	3	0	0

LGI, Lower gastrointestinal tract; GU, genitourinary. “Gy” means Gy (RBE).

The observed late toxicities are listed in Table 2C. One patient developed a Grade 4 sigmoid colon perforation in the phase 2 component, 24 months after treatment, which required a colostomy. The maximum dose to the sigmoid colon was estimated to be 55.2 Gy (RBE), which was outside PTV‐3. This patient underwent repeated laser coagulation for sigmoid colon bleeding before the perforation was detected. A second patient with peritonitis caused by appendicitis 16 months after treatment developed a Grade 3 small intestine complication 17 months after treatment. Upon laparotomy for the appendicitis, small intestine inflammation was detected. The dose to the small intestine was estimated to be between 36.0 and 55.2 Gy (RBE). These toxicities were reviewed and defined as Grade 4 or Grade 3, respectively, according to the Working Group of Gynecological Tumors.

### Treatment efficacy

Local tumor responses within 6 months of treatment at each total dose are displayed in Table 3. Twenty (80%) of the 25 patients who received the RD achieved a complete response (CR), with the remaining five patients (20%) achieving a partial response. There were no significant associations between the response rate and the dose delivered to PTV‐3.

**Table 3:** The tumor responses defined with RECIST criteria

		Tumor response			
Dose (Phase)	No.	CR	PR	SD	PD
68.0 Gy (Phase 1)	3	1	2	0	0
71.2 Gy (Phase 1)	3	2	1	0	0
74.4 Gy (Phase 1)	3	2	1	0	0
74.4 Gy (Phase 2)	22	18	4	0	0
No. of local recurrence (%)		5/23 (23%)	5/8 (60%)	N/A	N/A

N/A: not available.

**Figure 2:**
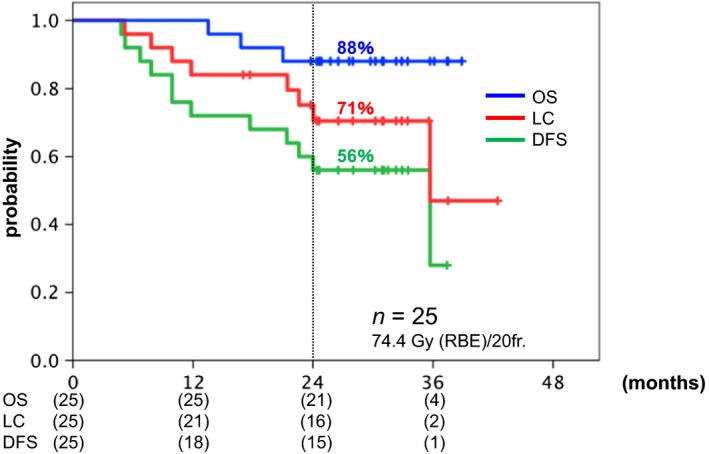
Kaplan–Meier curves of overall survival (OS; blue), local control (LC; red), and disease‐free survival (DFS; green) for the 25 patients treated with 74.4 Gy (relative biological effectiveness [RBE]). Number at risk is shown below the figure.

Ten patients developed local recurrence (LR) by the time of the last follow‐up (1 with 68.0 Gy [RBE], 1 with 71.2 Gy [RBE], and 8 with 74.4 Gy [RBE]). The clinical characteristics of the 10 patients with local recurrence are shown in Table S1. Five of the 10 patients who developed LR had undergone surgery, with recurrent tumors surgically salvaged in four of the five patients. The 2‐year DM rate was 32%. The 2‐year LC, DFS, and OS rates for all patients combined were 74%, 56%, and 84%, respectively (Fig.**** 1). In patients treated with the RD, the 2‐year LC, DFS, and OS rates were 71%, 56%, and 88%, respectively (Fig.**** 2). The 2‐year LC rates according to Stage IIB, IIIB, and IVA disease were 68%, 65%, and 100%, respectively (*P *>* *0.05).

## Discussion

This is the first study to evaluate the safety and efficacy of chemo‐C‐ion RT for locally advanced uterine cervical adenocarcinoma. To the best of our knowledge, this is also the first study to ascertain the feasibility of chemo‐C‐ion RT for the pelvic region. The optimal dose of chemo‐C‐ion RT for uterine cervical adenocarcinoma was unknown. As a result, no DLTs occurred in the dose range we set, even at a dose of 74.4 Gy (RBE) in 20 fractions, which was the highest dose in our previous dose escalation study 15 of C‐ion RT alone for locally advanced cervical adenocarcinoma. Considering the dose constraint of the GI, further dose escalation seemed hazardous. Thus, 74.4 Gy (RBE) in 20 fractions with concurrent weekly cisplatin (40 mg/m^2^) was considered the RD for the phase 2 component.

Regarding acute toxicities, only one patient developed Grade 3 nausea that required intravenous fluids. None of the remaining patients developed acute toxicities of Grade ≥3. Kirwan et al. [28] conducted a systematic review of acute and late toxicity of concurrent chemo‐RT for cervical cancer and reported that the incidence of acute hematological toxicities of Grade ≥3 was 0.6–27.6% for conventional CCRT. The lower incidence of acute toxicity in our study may be attributable to the excellent dose distribution of C‐ion RT, which reduces the dose to the bone marrow in the pelvic region (Fig. S2).

Only two patients who received the RD developed severe late GI toxicities. A previous randomized trial 6 revealed that 14% of patients developed Grade ≥3 late GI toxicities after CCRT for uterine cervical adenocarcinoma. Although the follow‐up duration is short and the number of patients is small, the incidence of severe late GI toxicities in this study seems to be comparable.

We previously reported on the relationship between GI toxicities and the dose of C‐ion RT to the rectum [29]. Based on these findings, the dose constraint to the GI was limited to <60.0 Gy (RBE) in this study. In the two patients who developed severe late toxicities, however, the maximum dose to the region where the adverse event occurred was <55.2 Gy (RBE). Both patients suffered injury to the GI tract, either through repeated laser coagulation or peritonitis caused by appendicitis. After chemo‐C‐ion RT, these injuries may have exacerbated the toxicity. However, the effect of chemo‐C‐ion RT cannot be denied; chemo‐C‐ion RT may indeed have a worse toxicity than standard RT including brachytherapy. Therefore, further follow‐up is needed to determine its long‐term safety.

Despite our study including patients with locally advanced Stage IIB‐IVA uterine cervical adenocarcinomas, good results were achieved, with a CR rate of 80% among patients who received the RD and a 2‐year LC rate of 74%. According to the correlation between LC and dose escalation, 10 patients developed LR in this study; 1 with 68.0 Gy (RBE), 1 with 71.2 Gy (RBE), and 8 with 74.4 Gy (RBE). However, the correlation between LC and dose escalation was not significant. Although mucinous adenocarcinoma and clear cell carcinoma showed relatively lower CR rates and higher LR rates than that of endometrioid adenocarcinoma and adenosquamous carcinoma, this difference was not statistically significant because of the small sample number. Thus, the correlation between LR and histological subtype needs to be further evaluated by studies using larger patient cohorts.

Indeed, RT or CCRT including intracavitary brachytherapy is considered the standard treatment for locally advanced uterine cervical adenocarcinoma. However, previous studies 14, 20, 30, 31, 32 have shown a poor prognosis, with 5‐year LC rates of just 33–58%. Meanwhile, we reported 15 that C‐ion RT alone exhibited comparable LC rates compared to those of conventional chemo‐RT, with 2‐ and 5‐year LC rates of 60% and 55%, respectively. In this study, we achieved a more favorable 2‐year LC rate of 74.0%. These favorable LC rates may be attributable to a biological advantage of C‐ion RT and a radiosensitizing effect of cisplatin to C‐ion beam irradiation. Recent in vitro studies 33, 34 have demonstrated that the addition of chemotherapeutic drugs to C‐ion RT is a potential method for enhancing its efficacy. Meanwhile, few studies have investigated the radiosensitizing effect of cisplatin to C‐ion beam irradiation in cervical cancer cell lines. Further research is still warranted to investigate the mechanisms of radiosensitization between cisplatin and C‐ion beam irradiation in human cervical cancer cell lines. Due to technological innovations in C‐ion RT, it is now possible to increase the dose to the tumor using C‐ion scanning beam therapy 35. Furthermore, a clinical trial consisting of C‐ion RT with brachytherapy for uterine cervical cancer is ongoing at another institution in Japan (UMIN000015015). These studies may contribute to better LC. In this study, the 2‐year OS rate was 84%. Several previous studies 14, 20, 31 have shown that 5‐year OS rates were just 20–60%. Our previous study 18 of C‐ion RT alone for cervical adenocarcinoma demonstrated 2‐ and 5‐year OS rates of 66% and 38%, respectively. Although the number of patients in this study is small, the OS rate was favorable compared to these studies. The poor OS rates of the previous studies are not only due to poor LC, but also a high frequency of DM. Huang et al. 36. reported a 5‐year DM rate of 46% for Stage III patients after RT alone or CCRT. Our previous study 18 of C‐ion RT alone for cervical adenocarcinoma revealed 2‐ and 5‐year DM rates of 49% and 65%, respectively. In contrast, the 2‐year DM rate of this study was 32%. Taken together, these findings suggest that chemo‐C‐ion RT not only reduces local failure, but also the DM rate of patients with uterine cervical adenocarcinoma.

In conclusion, chemo‐C‐ion RT was well tolerated in all but two patients with locally advanced uterine cervical cancer. Although the follow‐up period is short and the number of patients is small, we demonstrate the beneficial effects of chemo‐C‐ion RT in locally advanced uterine cervical adenocarcinoma. Our findings support further investigations into the therapeutic efficacy of chemo‐C‐ion RT.

## Conflict of Interest

The authors have no conflicts of interest to disclose.

## Supporting information


**Figure S1.** Isodose curves of carbon‐ion radiotherapy for uterus adenocarcinoma. (A) Sagittal and (B) axial computed cosmography images for the total irradiation plan with 74.4 Gy (RBE).

**Figure S2.** Isodose curves of (A) carbon‐ion radiotherapy and (B) X‐ray radiotherapy for uterus adenocarcinoma. Target volumes are highlighted in white. Carbon‐ion radiotherapy can reduce the dose in the middle‐ to low dose range to the pelvic bone marrow.

**Table S1**. The clinical characteristics of 10 patients with local recurrence.
**Table S2**. The tumor responses and local recurrences according to tumor histology.
