# Supplementary figures and images for: Clinical outcomes of carbon ion radiotherapy with concurrent chemotherapy for locally advanced uterine cervical adenocarcinoma in a phase 1/2 clinical trial (Protocol 1001)
Source: Cancer Med. 2018 Jan 17;7(2):351–9. doi: 10.1002/cam4.1305 (PMC5806111; doi:10.1002/cam4.1305)

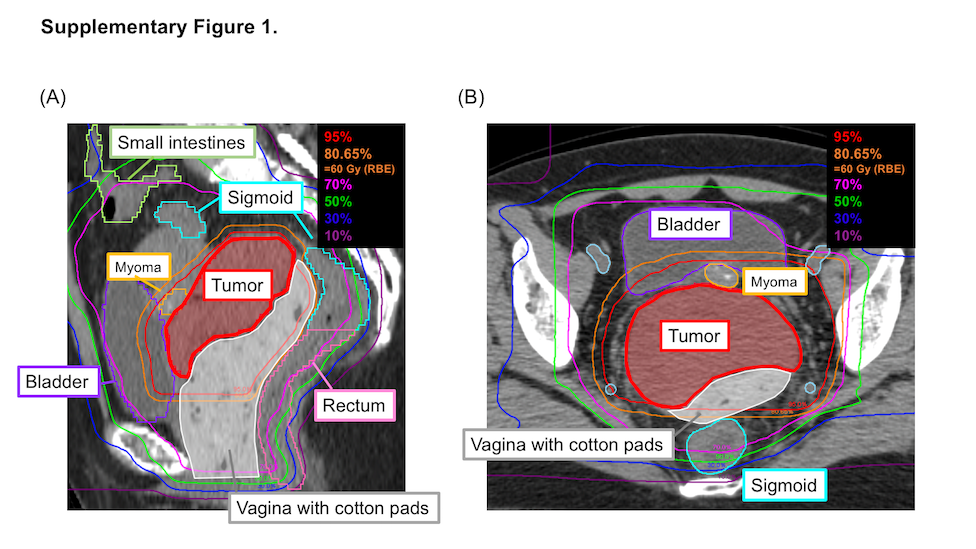

Supplement: Supplementary file 1 — Figure S1. Isodose curves of carbon‐ion radiotherapy for uterus adenocarcinoma. (A) Sagittal and (B) axial computed cosmography images for the total irradiation plan with 74.4 Gy (RBE). [file CAM4-7-351-s001.tiff]

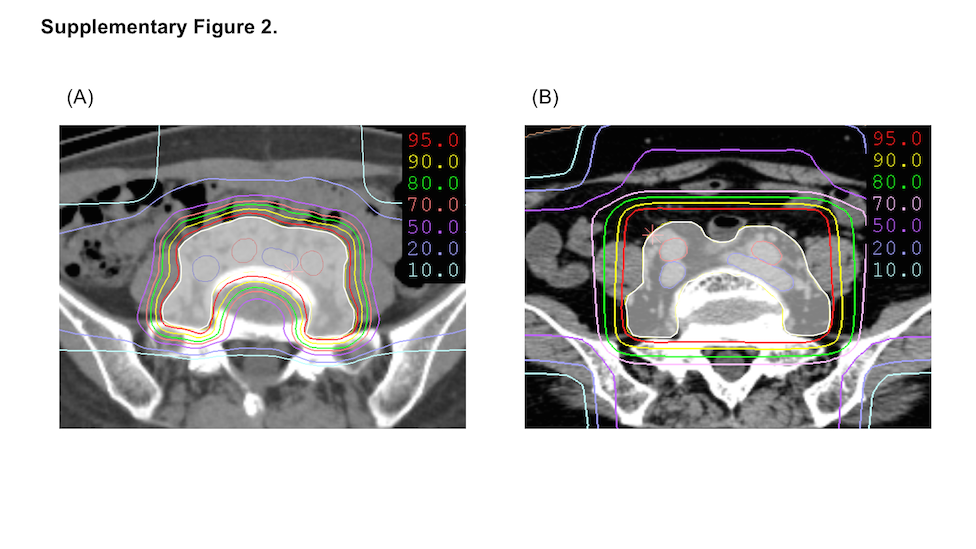

Supplement: Supplementary file 2 — Figure S2. Isodose curves of (A) carbon‐ion radiotherapy and (B) X‐ray radiotherapy for uterus adenocarcinoma. Target volumes are highlighted in white. Carbon‐ion radiotherapy can reduce the dose in the middle‐ to low dose range to the pelvic bone marrow. [file CAM4-7-351-s002.tiff]
