# Supplementary material for: Clinical outcomes of carbon ion radiotherapy with concurrent chemotherapy for locally advanced uterine cervical adenocarcinoma in a phase 1/2 clinical trial (Protocol 1001)
Source: Cancer Med. 2018 Jan 17;7(2):351–9. doi: 10.1002/cam4.1305 (PMC5806111; doi:10.1002/cam4.1305)
Supplement: Supplementary file 3 — Table S1. The clinical characteristics of 10 patients with local recurrence. Table S2. The tumor responses and local recurrences according to tumor histology. [file CAM4-7-351-s003.docx]

Supplementary Table 1. The clinical characteristics of 10 patients with local recurrence.

| TNM | Age | Histology | | C-ion RT  Dose  Gy (RBE) | | Tumor size (cm) | | | Response  at 6 months  in RECIST | | Periods from starting of the treatment (months) | |
| --- | --- | --- | --- | --- | --- | --- | --- | --- | --- | --- | --- | --- |
| cT3bN1N0 | 46 | | Mucinous | | 68.0 | | 7.6 | PR | | 7 | |  |
| cT2bN1N0 | 41 | | Mucinous | | 71.2 | | 6.2 | PR | | 7 | |  |
| cT2bN0N0 | 41 | | Mucinous | | 74.4 | | 4.7 | CR | | 12 | |  |
| cT2bN0N0 | 46 | | Clear cell | | 74.4 | | 7.1 | PR | | 7 | |  |
| cT2bN1N0 | 66 | | Clear cell | | 74.4 | | 4.4 | PR | | 10 | |  |
| cT2bN0N0 | 41 | | Mucinous | | 74.4 | | 5.8 | CR | | 36 | |  |
| cT3bN0N0 | 70 | | Mucinous | | 74.4 | | 3.5 | PR | | 8 | |  |
| cT3bN0N0 | 60 | | Mucinous | | 74.4 | | 6.3 | CR | | 23 | |  |
| cT2bN0N0 | 48 | | Mucinous | | 74.4 | | 4.2 | CR | | 24 | |  |
| cT2bN0N0 | 62 | | E Endometrioid | | 74.4 | | 3.5 | CR | | 21 | |  |

Abbreviation: RECIST, response evaluation criteria in solid tumors

Supplementary Table 2. The tumor responses and local recurrences according to tumor histology.

| **Histology** | **No.** | **Tumor response** | | **No. of**  **local recurrences (%)** |
| --- | --- | --- | --- | --- |
|  |  | **CR** | **PR** |  |
| **Mucinous adenocarcinoma** | 17 | 12 | 5 | 7 (41) |
| **Endometrioid adenocarcinoma** | 6 | 5 | 1 | 1 (17) |
| **Clear cell**  **carcinoma** | 3 | 1 | 2 | 2 (67) |
| **Adenosquamous carcinoma** | 5 | 5 | 0 | 0 (0) |

Abbreviation: CR: complete response, PR: partial response.
